# The Hormonal Effects of Alcohol Use on the Mother and Fetus

**Published:** 1998

**Authors:** Kara Gabriel, Candace Hofmann, Maria Glavas, Joanne Weinberg

**Affiliations:** Kara Gabriel, M.A., and Candace Hofmann, M.A., are doctoral students in the Department of Psychology and Maria Glavas, B.Sc., is a doctoral student in the Graduate Program in Neuroscience, University of British Columbia, Vancouver, British Columbia, Canada. All three students are pursuing their dissertation research in the laboratory of Joanne Weinberg, Ph.D. Joanne Weinberg, Ph.D., is a professor in the Department of Anatomy and an Honorary Professor in the Department of Psychology, University of British Columbia, Vancouver, British Columbia, Canada

**Keywords:** AODE (alcohol and other drug effects), gestation, mother, fetus, fetal development, polypeptide hormones, adverse drug effect, prenatal alcohol exposure, fetal alcohol syndrome, placenta, pituitary-adrenal axis, pituitary-thyroid axis, hypothalamus-pituitary axis, somatotropin, growth promoting factors, insulin, congenital anomaly, animal model, literature review

## Abstract

During pregnancy, the hormonal systems of the mother and fetus are intricately interconnected to ensure normal fetal development. Accordingly, maternal alcohol consumption during pregnancy can interfere with fetal development, not only directly, through adverse effects exerted by alcohol that crosses the placenta and enters the fetal bloodstream, but also indirectly, by disturbing the functions and interactions of maternal and fetal hormones. In both the mother and the fetus, alcohol exposure can impair the functioning of the hypothalamic-pituitary-adrenal axis, which regulates the body’s response to stress; the hypothalamic-pituitary-gonadal axis, which controls reproductive functions; and the hypothalamic-pituitary-thyroid axis, which regulates the metabolism of almost all tissues. In addition, alcohol can interfere with the activities of growth hormone and insulin-like growth factors, which promote body growth and activity. Some of the effects of maternal alcohol consumption on fetal hormone systems may contribute to the adverse effects observed in children with fetal alcohol syndrome and related disorders.

The endocrine (i.e., hormonal) system plays a critical role in maintaining the body’s internal equilibrium (i.e., homeostasis). Through the release of hormones, the endocrine system regulates functions as diverse as reproduction, stress response, metabolism, growth, and behavior. Two structures—(1) a brain region called the hypothalamus and (2) the pituitary gland, which is attached to the base of the brain through the pituitary stalk—work together to control the activity of most endocrine glands in the body, including the adrenal glands, gonads (i.e., ovaries and testes), and thyroid gland. In turn, each of those glands produces one or more hormones (e.g., cortisol, estrogen, testosterone, and thyroid hormone) that control various physiological activities in the body. Alcohol can alter the activities of all components of the endocrine system by acting either directly on the endocrine glands and/ or on the hypothalamus or pituitary.

In a pregnant woman, alcohol-induced alterations in endocrine activity may affect not only her health but also her ability to maintain a successful pregnancy. Moreover, alcohol consumption during pregnancy can directly affect fetal development because alcohol readily crosses the placenta. Finally, alcohol consumed during pregnancy may alter fetal development indirectly by disrupting the normal hormonal interactions between the mother and the fetus. This article reviews the multiple hormonal effects of alcohol use during pregnancy on the mother and fetus. The article incorporates findings from both human and animal studies, because although the amount of data from human subjects continues to grow, research using animal models of prenatal alcohol exposure has greatly expanded scientific understanding of alcohol’s effects on endocrine function.

## The Role of the Placenta

During pregnancy, the placenta plays a pivotal role in maintaining pregnancy and affecting fetal development. Until the fetal endocrine system is functional, the placenta acts as a miniature endocrine system, producing hormones such as human chorionic gonadotropin, chorionic thyrotropin, and chorionic corticotropin. Through its hormone production, the placenta regulates fetal growth, maturation, and nutrient utilization. In addition to its endocrine activity, the placenta acts as a partial barrier, or filter, between the maternal and fetal blood, allowing the transfer of some maternal hormones and other substances (e.g., alcohol) from the mother to the fetus while preventing the transfer of others. As a result of these placental functions, the fetus is exposed to three sets of hormones: (1) those secreted by the placenta, (2) those produced by the mother, and (3) the fetus’s own hormonal secretions.

Alterations in placental functioning, including the production and activity of placental hormones, may affect fetal growth and development and increase the risk of spontaneous abortion. To date, little information is available on the direct effects of alcohol on the placental hormones in humans. Animal models have extended researchers’ limited understanding of the placenta’s role in the effects of prenatal alcohol exposure.

## Alcohol’s Impact on Endocrine Functioning in Pregnant Women

Historically, alcohol research in humans has been conducted primarily in men. Only during the past two decades have researchers begun to analyze alcohol’s effects on women (for a review of alcohol’s effects on neuroendocrine function in women, see Mello et al. 1993). Studies have found that women have higher blood alcohol levels (BALs) than men after drinking the same amount of alcohol per body weight. In addition, women show an accelerated progression of alcohol-related adverse consequences, developing alcohol-induced liver disease and brain damage earlier in life and after lower alcohol intake than men (for a review, see Roman 1988). The reasons for the differential risks associated with alcohol consumption for women and men remain poorly understood, emphasizing the importance of further studies on alcohol’s impact on physiological functioning in women.

Even fewer studies have investigated the consequences of alcohol consumption during pregnancy on women’s physiological (including endocrine) functions. The maternal endocrine system undergoes numerous changes that are geared toward maintaining the pregnancy and providing support for the fetus. Any disruption of the maternal hormone balance can lead to poor pregnancy outcome, including fetal birth defects. As the following sections describe, alcohol can interfere with maternal endocrine functions through numerous mechanisms (also see textbox).

**Mechanisms of Alcohol- Induced Changes in Hormone Levels**

The concentration of a given hormone in the blood depends on many factors, including the rate at which the hormone is produced and secreted, its distribution in the body, and the rate at which it is removed from the blood. Alcohol can interfere with a hormone’s function by altering each of those factors. In addition, total hormone concentration does not necessarily correspond to the concentration of active hormone in the blood, because proteins called binding proteins, or globulins, may attach to and temporarily inactivate a hormone. Alcohol also may alter the levels of these proteins, thereby modifying endocrine activity and, during pregnancy, inducing complex consequences for both mother and fetus.

### Effects on the Hypothalamic-Pituitary-Adrenal Axis

The hypothalamic-pituitary-adrenal (HPA) axis is a hormone system that plays an essential role in the body’s response to stressful events (see table, p. 172). During periods of stress, the hypothalamus secretes corticotropin-releasing hormone (CRH), which, in turn, stimulates the release of adrenocorticotropic hormone (ACTH) from the pituitary gland. ACTH regulates the growth and activity of the outer layer of the adrenal glands (i.e., the adrenal cortex) and induces the secretion of adrenal hormones called glucocorticoids—cortisol (in humans) and corticosterone (in rodents). As the glucocorticoid levels increase in the blood, they act on the pituitary, hypothalamus, and other brain regions to inhibit further activation of the HPA axis. This process of inhibiting further hormonal activation is called negative feedback.

In the short term, glucocorticoids mobilize the body’s energy resources to respond to stress. This mobilization occurs at the expense of energy-dependent functions, such as digestion, growth, and reproduction. When the stressful situation is prolonged (i.e., lasts for weeks or months) or occurs frequently, the resulting metabolic effects and redistribution of resources may have pathological consequences, including the development of ulcers, growth retardation and underdevelopment (i.e., dwarfism) in children, and suppression of the immune system. Alcohol consumption also activates the HPA axis and stimulates glucocorticoid release, similar to the effects of stress. (For more information on alcohol’s effects on the HPA axis, also see the article by Gianoulakis, pp. 202–210).

The basal or resting activity of the HPA axis (i.e., the activity in the absence of stress) increases during pregnancy. This increase results in higher CRH and ACTH levels and, consequently, elevated glucocorticoid levels in the blood of pregnant females compared with nonpregnant females. Researchers have investigated alcohol’s impact on HPA activity in both pregnant and nonpregnant females. Studies of pregnant rodents found that alcohol consumption further stimulated an already activated HPA axis. Thus, compared with pregnant females on non-alcohol-containing diets, pregnant females receiving an alcohol-containing diet exhibited increases in adrenal gland weight, resting glucocorticoid levels, and the HPA response to stress (for a review, see Weinberg 1993). This alcohol-induced activation of the HPA axis occurred early during pregnancy and persisted throughout gestation, regardless of whether the alcohol-containing diet included high or low alcohol concentrations. Because the hormones of the HPA axis play numerous roles in energy distribution, metabolism, and immune function, alcohol-induced HPA activation may produce widespread physiological changes during pregnancy.

### Effects on the Hypothalamic-Pituitary-Gonadal Axis

The hormones of the hypothalamic-pituitary-gonadal (HPG) axis control reproductive functions and behavior. The HPG axis is activated by the secretion of gonadotropin-releasing hormone (GnRH) from the hypothalamus. GnRH, in turn, stimulates the release of luteinizing hormone (LH) and follicle-stimulating hormone (FSH) from the pituitary. Both LH and FSH regulate the development, growth, maturation, and reproductive functions of the gonads and stimulate the production of sex hormones, including estrogens and androgens (women also secrete small amounts of androgens, such as testosterone). Both estrogens and androgens activate numerous processes in the maturing organism, such as the onset of puberty, the development of secondary sex characteristics, and the behaviors associated with reproduction. In the developing fetus, androgens also have an organizing function, affecting not only the testes and ovaries but also the size and function of different brain regions.

**Table:** Hormone Systems Affected by Maternal Alcohol Consumption and Their Components

Hormone System or Hormone	Abbreviation	Site of Production
Hypothalamic-pituitary-adrenal axis (involved in the stress response)		
Corticotropin-releasing hormone	CRH	Hypothalamus
Adrenocorticotropic hormone	ACTH	Pituitary gland
Glucocorticoids		Adrenal glands
Cortisol (humans)		
Corticosterone (rats)		
Hypothalamic-pituitary-gonadal axis (controls reproductive functions and behavior)		
Gonadotropin-releasing hormone	GnRH	Hypothalamus
Luteinizing hormone	LH	Pituitary gland
Follicle-stimulating hormone	FSH	Pituitary gland
Estrogens		Primarily ovaries
Androgens (e.g., testosterone)		Primarily testes
Hypothalamic-pituitary-thyroid axis (controls overall metabolic rate in almost all tissues and organs)		
Thyrotropin-releasing hormone	TRH	Hypothalamus
Thyroid-stimulating hormone	TSH	Pituitary gland
Thyroxine	T_4_	Thyroid gland
Triiodothyronine	T_3_	Thyroid gland
Growth hormone system (promotes body growth and activity as well as energy storage in tissues)		
Growth hormone-releasing hormone	GHRH	Hypothalamus
Somatostatin	SS	Hypothalamus
Growth hormone	GH	Pituitary gland
Insulin-like growth factors 1 and 2	IGF-1, IGF-2	Primarily liver

Researchers have clearly documented the adverse effects of chronic alcohol consumption on women’s reproductive functioning. For example, alcoholic women have a higher frequency of menstrual abnormalities, such as irregular menstruation and the cessation of ovulation, than do nonalcoholic women (Mello et al. 1993). Those menstrual abnormalities may be mediated, at least in part, by alcohol-induced elevations in another pituitary hormone—prolactin—that suppresses ovulation (Mello et al. 1993).

Studies of alcohol’s effects on HPG activity in pregnant women found that alcohol use altered the levels of sex hormone-binding globulin (SHBG), a protein that binds to and temporarily inactivates androgens, thereby regulating the balance between biologically active and inactive androgens (Ylikorkala et al. 1988). During a normal pregnancy, SHBG levels increase with advancing gestational age. Ylikorkala and colleagues (1988) demonstrated that in heavy-drinking women whose alcohol abuse resulted in fetal damage, SHBG levels increased less than in nondrinking women. Perhaps as a result of this reduction in binding protein levels, the heavy-drinking women exhibited elevated levels of active testosterone between weeks 16 and 22 of gestation. These findings indicate that alcohol may affect androgen levels most strongly during the first half of pregnancy.

### Effects on the Hypothalamic-Pituitary-Thyroid Axis

The hypothalamic-pituitary-thyroid (HPT) axis regulates the rate of metabolism in the body and is essential for the normal growth and development of almost every organ system. The HPT axis is activated by the secretion of thyrotropin-releasing hormone (TRH) from the hypothalamus, which stimulates the release of thyroid-stimulating hormone (TSH) from the pituitary. TSH, in turn, regulates the growth and activity of the thyroid gland and the secretion of two structurally related thyroid hormones—thyroxine (T_4_) and triiodothyronine (T_3_). Of those two hormones, T_3_ is substantially more active, and much of the body’s T_4_ is converted into T_3_ in the liver and other tissues. Reductions in thyroid hormone levels, a condition called hypothyroidism, can result in serious consequences, the extent of which depends on the time during the person’s life when thyroid function becomes impaired.

In adults, hypothyroidism is marked by reductions in metabolic rate and energy expenditure, resulting in widespread changes in tissue function as well as in drowsiness and listlessness. In adult, nonpregnant humans, chronic alcohol abuse has long been associated with thyroid dysfunctions (Israel et al. 1979). Dysfunction of the thyroid most often results from alcohol-induced liver damage, which interferes with the conversion of T_4_ to T_3_. Research in pregnant animals has shown that alcohol consumption may reduce the levels of TSH in the blood but not in the pituitary, suggesting that alcohol alters maternal HPT activity (for a review, see Hannigan 1993). The functional significance of such alterations for both the pregnant female and the fetus, however, is still unknown.

### Effects on Growth Hormone and Insulin-Like Growth Factors

As the name implies, growth hormone promotes the body’s growth and activity as well as the storage of energy in various tissues, including fat (i.e., adipose), liver, muscle, bone, heart, and lungs. In the absence of growth hormone, both animals and humans show stunted growth. Conversely, the hormone’s presence results in increased growth as well as enhanced organ size and function. Growth hormone is produced in the pituitary gland. Secretion of the hormone is stimulated primarily by growth hormone-releasing hormone (GHRH), which is released from the hypothalamus. An inhibiting hormone that is also secreted by the hypothalamus—somatostatin—cooperates with GHRH to regulate growth hormone secretion. In addition, estrogens and androgens promote and glucocorticoids inhibit growth hormone release, demonstrating the interactive nature of the endocrine system.

Although growth hormone exerts some of its effects by acting directly on its target tissues, small proteins called insulin-like growth factors (IGFs), or somatomedins, mediate other effects. In adults, two forms of IGFs exist—IGF-1 and IGF-2—which are produced primarily in the liver. These growth factors, whose production is induced by growth hormone, act on various tissues to mediate the effects of growth hormone. In addition, IGFs are thought to play an important role in the regulation of fetal growth and development.

Studies in human alcoholics have not yet identified characteristic alcohol-induced alterations in growth hormone secretion and activity, at least in part because confounding factors (e.g., the duration of alcohol use and the presence of alcohol-associated illnesses) also influence growth hormone secretion. Animal research has shown that pregnant alcohol-consuming rats exhibit reduced growth hormone levels (for a review, see Weinberg 1993). In addition, several animal studies have indicated that alcohol consumption may alter IGF levels in pregnant females. For example, Breese and Sonntag (1995) reported that IGF-1 levels in the blood were reduced and IGF-2 levels in the blood were increased in alcohol-consuming pregnant rats compared with non-alcohol-consuming pregnant rats. Moreover, the researchers noted significant reductions in a component of IGF-binding protein, indicating that alcohol may alter the regulation of maternal IGF function. These alterations may contribute to overall changes in metabolism and other effects on both maternal and fetal systems.

## Alcohol’s Impact on Endocrine Functioning in the Fetus and Infant

Maternal alcohol consumption during pregnancy can produce devastating effects on the fetus. The most severe consequence is fetal alcohol syndrome (FAS), which is associated with characteristic patterns of abnormal facial structures, growth retardation, and developmental abnormalities of the central nervous system (CNS). FAS was first associated with maternal alcohol consumption more than 25 years ago (Lemoine et al. 1968; Jones and Smith 1973). More recently, researchers have adopted three terms to characterize children who were affected by alcohol prenatally but do not meet all the criteria for FAS (Stratton et al. 1996). The term “partial FAS” refers to children with confirmed prenatal alcohol exposure and evidence of some components of the characteristic facial anomalies but without full FAS. The term “alcohol-related birth defects” (ARBDs) is used for children who have primarily physical malformations or physiological abnormalities. Last, the term “alcohol-related neurodevelopmental disorder” (ARND) describes children with either physical CNS abnormalities (e.g., smaller head size or structural brain abnormalities) or with behavioral and/or cognitive abnormalities, such as deficits in memory, language skills, or learning ability.

The type and extent of the alcohol-induced fetal damage is partly related to the level and pattern of fetal alcohol exposure. For example, lower levels of prenatal alcohol exposure are required to induce neurodevelopmental effects than to induce physical or growth effects (Streissguth et al. 1989). Research in nonhuman primates has shown that behavioral deficits may occur without accompanying physical abnormalities (Clarren et al. 1990). Moreover, those studies found that effects of prenatal alcohol exposure are clearly observable after once-per-week alcohol consumption resulting in BALs above 140 milligrams per deciliter (mg/dL) (i.e., the equivalent of four to six standard drinks^1^ consumed by an average-sized woman). Indeed, maternal binge drinking (i.e., consumption of five or more standard drinks per occasion) during pregnancy is one of the strongest predictors of later neurodevelopmental deficits in children with alcohol-induced damage (Streissguth et al. 1989). Possibly, the effects of binge drinking are particularly severe because this drinking pattern results in high BALs in both mother and fetus, followed by repeated withdrawal episodes. Moreover, the fetus cannot metabolize alcohol effectively, because its immature liver does not produce the necessary enzymes.

The type of fetal damage that results may also be related to the timing of alcohol exposure (i.e., whether the fetus is exposed to alcohol during the critical period of development of a particular organ system). The critical period is the time during which an organ system is undergoing crucial steps in development and/or maturation and consequently is most vulnerable to the disruptive effects of any agent that causes abnormal fetal development or birth defects. For example, because the development of the facial and skull bones occurs during the first 3 months of gestation in humans, alcohol exposure during the first trimester can affect those structures and result in the characteristic facial abnormalities observed in children with FAS. Conversely, alcohol exposure during the second or third trimester is more often associated with growth retardation and neurological defects, because fetal growth and brain development happen more rapidly during those gestational stages. If maternal alcohol consumption occurs during all three trimesters, the fetus is exposed to alcohol during the critical periods for the development of facial characteristics, growth patterns, and CNS function and thus may develop full FAS.

Prenatal alcohol exposure may adversely affect the fetal endocrine system and, consequently, the functioning of numerous organ systems. The endocrine activities of both mother and fetus change throughout gestation. For example, whereas the transfer of maternal hormones across the placenta and/or placental hormone production is essential during early pregnancy, the activity of the fetal endocrine system becomes more pronounced and important later in gestation. Consequently, alcohol’s impact on fetal endocrine activity may occur through different avenues at different time periods. Because of their numerous effects on physiological processes, alcohol-induced alterations in hormone levels likely mediate some of the effects of prenatal alcohol exposure.

### Effects on the HPA Axis

The HPA axis is essential for life because it affects the metabolism and activity of numerous systems (e.g., the nervous system and the immune system) and ensures homeostasis in response to stress. In the fetus, the first cells to become functional in the pituitary are those that release ACTH. By 9 weeks of gestation, the human fetal pituitary gland contains measurable ACTH levels. Because maternal ACTH cannot cross the placenta into the fetal circulation, the fetal HPA system is controlled by a placental hormone called human chorionic corticotropin until the fetal hypothalamus and pituitary have fully matured. Maternal glucocorticoids (e.g., cortisol), however, can cross the placenta and enter the fetal circulation.

As noted previously, alcohol consumption activates the HPA axis and stimulates glucocorticoid release. Therefore, alcohol consumed during pregnancy will activate the maternal HPA axis and result in increased glucocorticoid levels. Those glucocorticoids can cross the placenta, resulting in elevated glucocorticoid levels in the fetal blood, thereby signaling the fetal HPA axis to decrease its activity. At the same time, however, alcohol in the maternal blood also crosses the placenta and directly activates the fetal HPA axis. Such conflicting messages may alter the development of the fetal HPA axis by disrupting communication among the CNS, hypothalamus, pituitary, and adrenal glands.

To investigate alcohol’s effects on the fetal HPA axis, researchers have compared HPA activity in newborn rodents that were prenatally exposed to alcohol with newborn control animals that were not exposed to alcohol. In those studies, alcohol-exposed neonates showed evidence of prenatal HPA activation, such as elevated corticosterone levels in the blood and brain for several days after birth (see Weinberg 1993). Thereafter and up to approximately 3 weeks of age, the alcohol-exposed animals exhibited a reduced HPA response to various kinds of stress (e.g., exposure to a novel environment, cold, or ether), as indicated by reduced levels of corticosterone and β-endorphin^2^ (see Weinberg 1993). Those findings suggest that prenatal alcohol exposure may delay the maturation of the HPA axis or of the pathways in the brain that activate the hypothalamus in response to stress.

After the first few weeks of life, the pattern of reduced responsiveness to stress is reversed. In fact, adolescent and adult animals prenatally exposed to alcohol typically display increased HPA activation in response to various types of stress as indicated by the following observations (see Weinberg 1993).

- In response to stress such as immobilization, ether, or cold, alcohol-exposed animals show elevated corticosterone, ACTH, and/or β-endorphin levels compared with control animals.
- Animals prenatally exposed to alcohol show increased ACTH and corticosterone levels following one-time (i.e., acute) exposure to drugs such as alcohol and morphine compared with control animals.
- Stress-induced elevations in ACTH and corticosterone levels appear to be prolonged in alcohol-exposed animals following immobilization stress, suggesting that those animals may be less able to inhibit HPA activity or recover from stress than control animals.

Excessive HPA activity may have profound consequences throughout the animal’s life, including possible impairments in growth or immunity resulting from a redistribution of energy resources. Interestingly, the effects of prenatal alcohol exposure may differ between male and female offspring and may also depend on the nature and intensity of the stressor applied, the hormone analyzed (e.g., corticosterone, ACTH, or β-endorphin), and the time course of hormone measurement. For example, when animals were exposed to repeated immobilization stress, males prenatally exposed to alcohol showed increases in β-endorphin levels, whereas females showed increases in ACTH levels. In contrast, control males and females showed habituation (i.e., lower hormone levels) in their HPA responses to this repeated, predictable stressor (Weinberg et al. 1996). This example emphasizes not only that alcohol-exposed males and females may respond differently to stress but also highlights that alcohol-exposed animals may show deficits in their ability to use or respond to cues in their environment.

Similar to the findings in animals, a recent study in human infants whose mothers drank heavily at conception found greater increases in cortisol levels in response to stress (e.g., having blood drawn) compared with control infants (Jacobson et al. in press). Furthermore, children prenatally exposed to alcohol are known to be hyperactive, uninhibited, and impulsive in behavior, particularly in challenging or stressful situations. Because the hormones of the HPA axis act on the CNS to alter behavior and performance in stressful situations, altered HPA activity may underlie some of the behavioral problems seen in children prenatally exposed to alcohol.

### Effects on the HPG Axis

During gestation, the HPG axis influences not only the development of the reproductive system but also the organization of the CNS.^3^ Under the influence of testosterone (and, possibly, estrogen), certain brain areas (i.e., sexually dimorphic areas) develop differently in males and females. For example, several areas in the brain differ in size between males and females. In particular, the preoptic area of the hypothalamus contains a region—the sexually dimorphic nucleus of the preoptic area (SDN–POA)—that is considerably larger in males than in females and plays an important role in sexual and maternal behaviors in rats (Arnold and Gorski 1984).

Research conducted in animal models has shown that prenatal alcohol exposure adversely affects male offspring by altering the structure and function of the fetal testes as well as other components of the urogenital tract (see Weinberg 1993; McGivern and Riley 1993). For example:

- In the fetus, alcohol decreases the number of testosterone-producing cells in the testes and reduces testosterone production by inhibiting certain enzymes.
- At birth, animals prenatally exposed to alcohol exhibit decreased brain levels of the active form of testosterone (i.e., dihydrotestosterone) and decreased testosterone levels in the blood compared with control animals.
- Adult male animals prenatally exposed to alcohol exhibit lower testis and prostate weights as well as decreased levels of testosterone and LH (which is released from the pituitary to stimulate testosterone formation in the testes).

Alcohol-induced reductions in testosterone levels may alter testosterone’s effects on brain organization during gestation and may explain the differences in brain structure and sex-typical behavior seen between animals prenatally exposed to alcohol and control animals. For example, the SDN–POA in the hypothalamus is smaller in males prenatally exposed to alcohol than in control males (Barron et al. 1988). These alcohol-induced structural alterations may contribute to the changes in sex-typical behaviors observed after prenatal alcohol exposure. For example, compared with control animals, male rats prenatally exposed to alcohol take longer to mount females during copulation and show a decreased frequency of intromission (i.e., penetration) (Udani et al. 1985). Another sex-typical behavior is found in the navigation of a complex, spatial maze (i.e., the Lashley III maze), which male rats typically learn faster than do female rats. Male rats prenatally exposed to alcohol, however, require more trials to learn the maze than do control males, whereas female rats prenatally exposed to alcohol require fewer trials to learn the maze than do control females (McGivern and Riley 1993). These data suggest that at least in rats, prenatal alcohol exposure may result in the “feminization” of behavior in males and in the “masculinization” of behavior in females.

Changes in HPG activity and in behaviors controlled by the HPG axis also have been noted in female rodents prenatally exposed to alcohol, including the following (see Weinberg 1993; McGivern and Riley 1993):

- Increased levels of the pituitary hormone prolactin and decreased levels of LH in the blood from approximately 22 to 35 days of age (the elevated prolactin levels may persist into adulthood)
- Delays in puberty and in displaying sexually receptive behavior, possibly resulting from the hormonal changes
- Impaired maternal behavior (e.g., nest building and pup retrieval)
- Deficits in sexual behavior and earlier cessation of estrus cycling (i.e., the equivalent to menopause in humans), indicating that prenatal alcohol exposure may accelerate the aging process of the female reproductive system (McGivern et al. 1995).

Limited data are available on the association between prenatal alcohol exposure and HPG activity in humans. The available information indicates that prenatal alcohol exposure may slightly delay puberty in males, although the time of onset of puberty still is generally within normal limits in those adolescents (Streissguth et al. 1991). More extensive investigations regarding the effects of prenatal alcohol exposure on HPG activity and sexual development and behavior in humans are needed.

### Effects on the HPT Axis

Normal functioning of the HPT axis is critical for growth and development as well as for regulation of the body’s overall metabolic rate. Reductions in thyroid hormones during the first 2 years of life may be particularly devastating, because those hormones play a crucial role in CNS development. Accordingly, untreated hypothyroidism in infancy results in growth retardation (i.e., short stature) and mental retardation. During the first weeks of gestation, the transfer of maternal thyroid hormones to the fetus may play an important role in fetal development. By 11 to 12 weeks of gestation, however, the thyroid of the human fetus can produce and secrete its own hormones. Maternal alcohol consumption may reduce the availability of thyroid hormones to the fetus either indirectly, by inhibiting the transport of maternal hormone across the placenta, or directly, by interfering with the function of the fetal thyroid once it is active.

Studies in animal models have demonstrated that following prenatal alcohol exposure, the growth of the thyroid gland is retarded (for a review, see Hannigan 1993). For example, in rodents prenatally exposed to alcohol, reduced thyroid hormone levels were observed during the first 2 to 4 weeks of postnatal life. Researchers also have noted that numerous parallels exist between the effects of prenatal alcohol exposure and hypothyroidism. For example, in both conditions the cell numbers in certain brain regions that are important for memory (i.e., the hippocampus) and motor control (i.e., the cerebellum) are reduced. Furthermore, both conditions result in fetal growth retardation; delayed skeletal maturation; and behavioral problems, such as hyperactivity and learning disabilities. Finally, researchers have found that treatment with thyroid hormone soon after birth reversed some of the developmental delays observed in animals prenatally exposed to alcohol (Gottesfeld and Silverman 1990), further supporting the association between prenatal alcohol exposure and hypothyroidism.

An analysis of numerous studies on children prenatally exposed to alcohol, however, showed no long-term changes in thyroid function or thyroid hormone levels (Hannigan et al. 1995). The discrepancy between the findings in humans and those in animals may be partially attributable to differences between humans and animals in the critical periods during which alcohol exposure occurs. For example, rodents typically are tested for HPT function soon after birth, a time that is developmentally equivalent to the third trimester of human gestation. Therefore, effects of prenatal alcohol exposure on HPT function that are detectable after birth in rodents may occur during the last trimester of gestation in humans and may have already recovered before the infants are assessed after birth. In addition, the alcohol-induced changes in the regulatory and/or growth-promoting roles of thyroid hormones during fetal development may be more subtle in human newborns than in rats. Nevertheless, it is possible that even mild alcohol-induced prenatal alterations in HPT activity could have lasting effects on the function of many body systems and might contribute to the adverse effects of fetal alcohol exposure.

### Effects on Growth Hormone and IGFs

Growth hormone and IGFs play a central role in promoting body growth and development. Accordingly, alcohol-induced impairment of this hormone system during fetal development could have severe consequences. Animal studies have shown that newborn animals prenatally exposed to alcohol had reduced growth hormone levels compared with control animals (Thadani and Schanberg 1979). More recently, researchers have shown that the activity of IGFs, which play an essential role in fetal development, may be affected by prenatal alcohol exposure. Compared with control animals, young animals that had been prenatally exposed to alcohol exhibited lower IGF-1 levels in the blood and brain as well as reduced expression of the genes responsible for the production of both IGF-1 and IGF-2 in the liver (Singh et al. 1996; Mauceri et al. 1996). Both the diminished fetal brain concentrations of IGF-1 and the decreased expression of the IGF genes may contribute substantially to the retardation of brain growth associated with FAS. Interestingly, prenatal alcohol exposure appears to result in increased expression of the gene for IGF-binding protein in the fetal lung and placenta (Fatayerji et al. 1996). Increased levels of IGF-binding protein may decrease the levels of active IGFs in those tissues, thereby contributing to general fetal growth retardation.

Growth hormone normally is released from the pituitary periodically throughout the day. Growth hormone release increases in response to various factors, such as changes in blood sugar or insulin levels, fasting, or exercise. In children with FAS, growth hormone responses to stimulation by such factors were normal, as were growth hormone levels during sleep (for a review, see Rudeen and Taylor 1992). In contrast to those results from growth hormone stimulation tests, the estimated rate of spontaneous 24-hour growth hormone secretion in children with FAS was lower than in children of normal stature and similar to that of children who were born small for their gestational age (Hellstrom et al. 1996). In addition, the concentrations of IGF-1 and of one component of IGF-binding protein in the blood of children with FAS were at the lower end of the normal range (Hellstrom et al. 1996). These observations suggest that subtle alterations in growth hormone or IGF levels may contribute to alcohol-induced growth retardation.

## Conclusions

Alcohol consumption during pregnancy disrupts the normal functioning of both the maternal and the fetal endocrine systems and may disturb the normal maternal-fetal endocrine balance. Those alterations may adversely affect the development and organization of multiple systems in the fetus and likely mediate some commonly observed effects of pre-natal alcohol exposure. The exact mechanisms underlying alcohol-induced fetal damage have not been fully delineated. The tremendous impact of prenatal alcohol exposure on fetal development is not surprising, however, considering the interrelationship of the maternal and fetal endocrine systems, the complex role of the placenta, and the numerous direct and indirect effects of maternal alcohol consumption on both the mother and the fetus. Thus, although numerous factors likely play a role in alcohol-induced fetal damage, the disruption of hormonal influences on the developing fetus may explain at least some of the effects of prenatal alcohol exposure. An increased understanding of endocrine function during pregnancy; of the development of the fetal endocrine system; and of the processes influenced by maternal, placental, and fetal hormones may offer new insights into the adverse effects of prenatal alcohol exposure and possible methods of attenuating those effects.

## Footnotes

1 One standard drink is defined as one 12-ounce can of beer or wine cooler, one 5-ounce glass of wine, or 1.5 ounces of distilled spirits.

2 β-Endorphin is a peptide similar to morphine that is produced in the brain and other organs. It is released by the pituitary in response to stress.

3 In humans, the external genitalia of both sexes begin to develop at 9 to 10 weeks after conception, and the gender-specific differentiation of the brain occurs throughout gestation. In rodents, the process of sexual differentiation occurs in the last week of pregnancy and continues during the first 2 weeks after birth.
